# COVID-19 booster prioritization in the West Bank: a survey experiment among Bedouins, refugees, and the majority group

**DOI:** 10.3389/fpubh.2023.1227559

**Published:** 2023-10-04

**Authors:** Sarah Carol, Ahmad Amro

**Affiliations:** ^1^School of Sociology, University College Dublin, Dublin, Ireland; ^2^Social Science Research Center Berlin, Berlin, Germany; ^3^Faculty of Pharmacy, Al-Quds University, Jerusalem, Palestine

**Keywords:** COVID-19, vaccination, West Bank, solidarity, religion, intergroup attitudes

## Abstract

**Introduction:**

Our main aim is to understand to what extent Bedouins, internally displaced Palestinians (refugees) and majority-group members (non-refugees, non-Bedouins, settled) in the West Bank prioritize COVID-19 booster shots for their own group over other groups.

**Methods:**

We conducted a survey experiment (face-to-face) among 678 Palestinians living in the West Bank. Participants randomly received a description of an older man (Bedouin, refugee, settled) and were asked to indicate to what extent this person should be prioritized for the booster shot. Respondents belonging to a minority saw the profile of an in-group member or a majority-group member, whereas majority-group members would see the profile of an in-group or one out-group member (Bedouin, Palestinian refugee).

**Results:**

We found slightly higher in-group preferences for Palestinian refugees when it came to vaccination, whereas majority-group members were less inclined to support a prioritization of Palestinian refugees but equally prioritized their group and Bedouins. For Bedouins, we did not find strong in-group preferences.

**Discussion:**

Our study reveals the salience of group boundaries during the COVID-19 pandemic with potentially adverse effects on the health care of minorities.

## Introduction

Across the globe, we saw an unequal access to vaccines. On top of this disadvantage that many countries in the Global South experience, comes inequality in the prioritization for vaccination, potentially placing minorities within these countries lower in the queue. The vast amount of studies did not center on minorities when investigating vaccination priorities [e.g., (1–4)]. Yet, previous articles have highlighted the relevance of prioritizing vulnerable groups such as refugees, as overcrowded living conditions accelerate the spread of COVID-19 (5). However, is this view also supported within the population? When resources are scarce, such as a shortage in medical services and vaccines, the question of eligibility arises. A newly introduced term for this is vaccine chauvinism. The concept of vaccine chauvinism is derived from the concept of welfare chauvinism, which describes the idea that a group sees its members as more eligible for these resources. In the context of migration, those who have contributed to the welfare state for a longer time and/or have contributed more (mostly majority-group members) perceive themselves to be also more entitled to welfare support compared to immigrants (6). However, in this case, these perceived differences in deservingness concern vaccination, rather than welfare support more generally.

We argue that the extent to which individuals display vaccine chauvinism will depend on the group belonging. We hypothesize that the majority displays higher levels of in-group favoritism, as they have on average a higher social status than minorities. Minorities are more often deprived and might have therefore contributed less to the welfare system. Previous research has shown that in-group and out-group boundaries in terms of national belonging were reinforced during the pandemic when it came to pro-social intentions (7, 8). Along these lines, immigrants were generally prioritized less (6, 9).

But does this also extend to native minorities? This is an interesting question, as native minorities are citizens, but we further argue that salient group boundaries oftentimes go beyond citizenship. Native minorities across the world struggle with equal rights and accommodation [e.g., (10)]. A non-experimental study in the US revealed prioritization of Black, Hispanic, Native American, and other communities that have been disproportionately affected by COVID-19 (11). A follow-up study discovered that this was conditional on the risk status. A slight majority would toss a coin if the minority and non-minority member had an identical risk of severe COVID-19 (12).

To gauge the risk people are exposed to, we take into consideration if they classify themselves as belonging to the at-risk group, their previous vaccinations and deaths related to COVID-19 within their networks. A high number of deaths related to COVID-19 within one’s networks suggests increased exposure to the virus. In addition to group belonging based on migration experience and minority status, we also investigate the role of religiosity in vaccine chauvinism. Several studies have suggested that more religious individuals tend to hold more pro-social attitudes (13, 14), as they stress creed of brotherhood (15). Hence, more religious individuals regardless of their religion should therefore be more willing to prioritize others. However, prior research on the relationship between religiosity or religious affiliation on the one hand and attitudes toward out-groups on the other hand also revealed a negative relationship (16–19).

To understand vaccine chauvinism and individuals’ preferences for prioritization in COVID-19 vaccination, we conducted a survey experiment among 678 respondents living in the West Bank (area A under the Palestinian Authority). Our sample is composed of Bedouins, internally displaced individuals (in the following abbreviated as refugees) and majority-group members (non-refugees and non-Bedouins). Survey experiments provide a unique opportunity to approximate average causal effects of how belonging to a group influences attitudes toward other groups. While those experiments have been implemented in the Western hemisphere [e.g., (6)], they are very rare in countries shaken by instability in health care provision, and to the best of our knowledge focus more on immigrants than native minorities. Hence, our study attempts to fill a research gap.

The West Bank constitutes a highly relevant case, as the responsibility for health care is divided between the Palestinian Authority, Israel and the United Nations Relief and Works Agency for Palestine Refugees in the Near East (UNWRA), which complicates health care services. Existing studies point out the deficits in health care infrastructures (20). We focus on Bedouins and refugees, as they belong to the biggest native minorities in the West Bank and are one of the most vulnerable populations in Palestine (21). Palestinian refugees in the 19 camps within the West Bank are supported almost exclusively by the UNWRA, as their legal status means they either cannot access government health care or cannot afford to pay for alternatives (22). Access to health care and medication is said to have worsened throughout the pandemic for all groups in the West Bank, but particularly for Bedouins and Palestinian refugees (23). Living in overcrowded camps, Palestinian refugees contracted COVID-19 more often than other groups (24). Yet, they did not belong to the prioritized groups for vaccination. As in other countries, medical staff, chronically ill, and older people were prioritized (25). However, non-representative online surveys indicate a relatively high willingness to get vaccinated (26), but the actual numbers are low (27). This speaks to other studies suggesting a lower willingness among health-care workers to get vaccinated (28). About one third is estimated to have received a vaccine at the time of our survey (29).

By presenting novel data on a research context and a research design less frequently covered in public health, we contribute to the existing state-of-the-art. Our main argument is that group boundaries are a salient factor when investigating health care and vaccination. If groups have strong perceptions of ‘us’ vs. ‘them’, these boundaries will be stronger and will explain why individuals do not support the health care of minorities. The study can inform us beyond COVID-19, as findings also bear implications for minorities’ health care more generally.

## Data and methods

### Participants

The Palestinian population is estimated to be 5.35 million (30). The number of Bedouins is approximately 40,000 people (21). The population size of the refugee camps is based on estimates from UNRWA with the following numbers (West Bank Field Monitoring and Evaluation Office 2022): Alfawar (12,203), Dheisheh (18,558), Aqbat Jaber (10,033), Alamari (14,882), and Balata (31,791).

In our data, we analyzed 678 valid cases that were collected between October 2021 and February 2022. Among those are 125 Bedouins from the Jahaleen tribe (east of Jerusalem), Ka’abneh tribe (Jordan valley), and Rashaydeh tribe (southeast of Bethlehem) (31), 210 internally displaced individuals from the West Bank (from the Alfawar, Dheisheh, Aqbat Jaber, Alamari, and Balata camps), and 343 Palestinians who are neither Bedouins nor refugees and are called majority-group members in this study. We employed community-based sampling to gain access to the camps and tribes. Fieldworkers and volunteers from the Palestinian refugee camps and Bedouin tribes were trained and conducted pretests. At the time of the survey, fieldworkers were holding a Master or a doctoral degree. Volunteers are university students in their third or last year and all of them were supervised by the project leader. For the data collection, the team rented a car and visited the camps along with paper and pencil questionnaires, which were later manually entered. Respondents were interviewed in standard Arabic. Participants provided written consent. We incentivized the more vulnerable and hard-to-reach-populations of Bedouins and internally displaced Palestinians with a small amount (approximately €2,50).

### Tools

Each respondent randomly saw one of those scenarios where the group belonging was signaled. Each minority group saw the description of a man belonging to their group or the majority group, whereas majority-group members also saw the profiles of a Palestinian refugee and Bedouin in addition to their own group. The treatment was balanced, and profiles nearly equally distributed within groups, as Table 1 illustrates.

**Table 1:** Sample characteristics.

Total		Majority					Refugees					Bedouins				
	n	n	Mean	p50	SD	IQR	n	Mean	p50	SD	IQR	n	Mean	p50	SD	IQR
*Dependent variable*																
Prioritization	678	343	3.19	4	1.16	1	210	2.99^+^	3	1.22	2	125	2.77***	3	1.22	2
*Age*	567	281	24.9	20	13.3	4	178	26.5	22	10.7	8	108	32.0***	28	12.7	18
*Religiosity*	605	301	6.68	7	2.37	4	185	6.66	7	2.17	3	119	6.51	7	2.10	3
*Perceived discrimination*	678	343	1.45	0	2.41	2	210	1.95*	1	2.73	3	125	3.22***	4	2.59	5
	n	n	%				n	%				n	%			
*Treatment*	678	343					210					125				
Profile Majority			34					49					50			
Profile Bedouin			31					0					50			
Profile Refugee			35					51					0			
*Education*	651	327					204					120				
No education			2					5					6^+^			
Elementary School			5					1^+^					14***			
Secondary School			19					10*					34***			
Vocational training			5					14**					13**			
Bachelor			62					64					28***			
Master			5					5					3			
Doctorate			2					0					1			
*Female*	636	312	57				201	49^+^				123	33***			
*COVID-related variables*																
Fully vaccinated	609	303	77				191	73				115	80			
At risk	524	276	58				156	60				92	60			
COVID-related deaths	577	292	67				181	75				104	66			
*Sunni*	658	329	83				204	96***				125	95***			

Significant differences compared to majority-group members, ^+^*p* < 0.10, ^*^p < 0.05, ^**^p < 0.01, ^***^p < 0.001.

p50 (Median), IQR (Interquartile Range), SD (Standard Deviation).

*The outbreak of COVID-19 has placed an immense burden on societies and individuals who have become more isolated. Over the past months, countries have started to vaccinate its population against COVID-19. However, vaccines against COVID-19 are more effective with a booster shot. Imagine the case of a 70-year old* [*man who is a Palestinian refugee* (Palestinian refugee, majority)/*Bedouin man* (Bedouin, majority)/*Palestinian man who has lived in his house his entire life* (all groups)]*. Do you think that he should be prioritized when the vaccination booster shots are given? Please answer on a scale from 1 “Disagree strongly” to 5 “Agree strongly.”*

In an additional analysis, we include a number of socio-demographic control variables (Table 2, Model 2) such as *sex* (0 “male,” 1 “female”), *education* (0 “no education,” 1 “Elementary School,” 2” Secondary School,” 3 “Vocational Training,” 4 “Bachelor,” 5 “Master,” 6 “Doctorate”). In addition, we controlled if participants have been fully vaccinated, if they count as an at-risk group for COVID-19-complications, and if they knew anyone who died of *COVID*–19 in person (0 “no,” 1 “yes”). *Religiosity* was measured with the question “Regardless of whether you belong to a particular religion, how religious would you say you are on a scale from 0 “not religious at all” to 10 “very religious”?” Respondents were also asked what their religious denomination is. We recoded the variable to distinguish only between Sunni Muslims (0) and non-Muslims (Christians/others) (1). Given the smaller sample sizes, we merged Catholic, Protestant and Christian Orthodox into one category, and added those who categorized themselves as none’s or other religion (7 respondents). Perceived *discrimination* was an additive index, adding up the number of discrimination experiences (0 “no,” 1 “yes”) in different places of public life (Shops, bank or restaurant; Public areas such as parks and streets; Internet, social media; Work, job market; Public transport or taxis; School; Police; Housing; Courts; Border; Health care). Table 1 displays the descriptive statistics for these variables.

**Table 2:** Deservingness of booster shot (ordinary least squares regression).

	(1)	(2)
*Interactions group # profile*		
Majority ➔ Majority (ref.)		
Refugees ➔ Majority	−0.443**	−0.425**
	(0.161)	(0.163)
Bedouins ➔ Majority	−0.435*	−0.345+
	(0.185)	(0.198)
Majority ➔ Bedouin	0.0297	0.157
	(0.158)	(0.158)
Majority ➔ Refugee	−0.267+	−0.0920
	(0.155)	(0.158)
Refugees ➔ Refugees	0.573*	0.345
	(0.225)	(0.230)
Bedouins ➔ Bedouins	−0.177	−0.289
	(0.265)	(0.264)
*Education*		
No education (ref.)		
Elementary School		−0.284
		(0.308)
Secondary School		0.143
		(0.264)
Vocational training		0.537+
		(0.288)
Bachelor		0.271
		(0.254)
Master		0.176
		(0.318)
Doctorate		−0.132
		(0.469)
*Age (centered)*		0.0115*
		(0.00492)
*Female*		−0.0182
		(0.0963)
*Perceived discrimination*		−0.0417*
		(0.0185)
*Fully vaccinated*		0.131
		(0.110)
*At risk*		0.195+
		(0.117)
*COVID-related death in network*		0.0585
		(0.106)
*Religion*		
Religiosity		0.0474+
		(0.0248)
Non-Muslims (Christian/others) vs. Sunni Muslims		−0.437**
		(0.167)
Constant	3.276***	2.555***
	(0.110)	(0.319)
Observations	678	678

Standard errors in parentheses, + *p* < 0.10, * *p* < 0.05, ** *p* < 0.01, *** *p* < 0.001.

### Statistical analyses

To obtain estimates, we ran ordinary least square regressions in STATA. Model 1 (Table 2) contains estimates from the net model including only the profile respondents saw interacted with their group belonging (majority, Bedouin, refugee). In a subsequent step (Table 2, Model 2), we include control variables. Some of these variables contain missing values (due to refusal or not knowing an answer). Missing values were replaced after 20 multiple imputations using Markov Chain Monte Carlo. *AIC* values to compare the quality of models are not displayed, as the criterion cannot be estimated for the model with imputed values.

## Results

**Figure 1:**
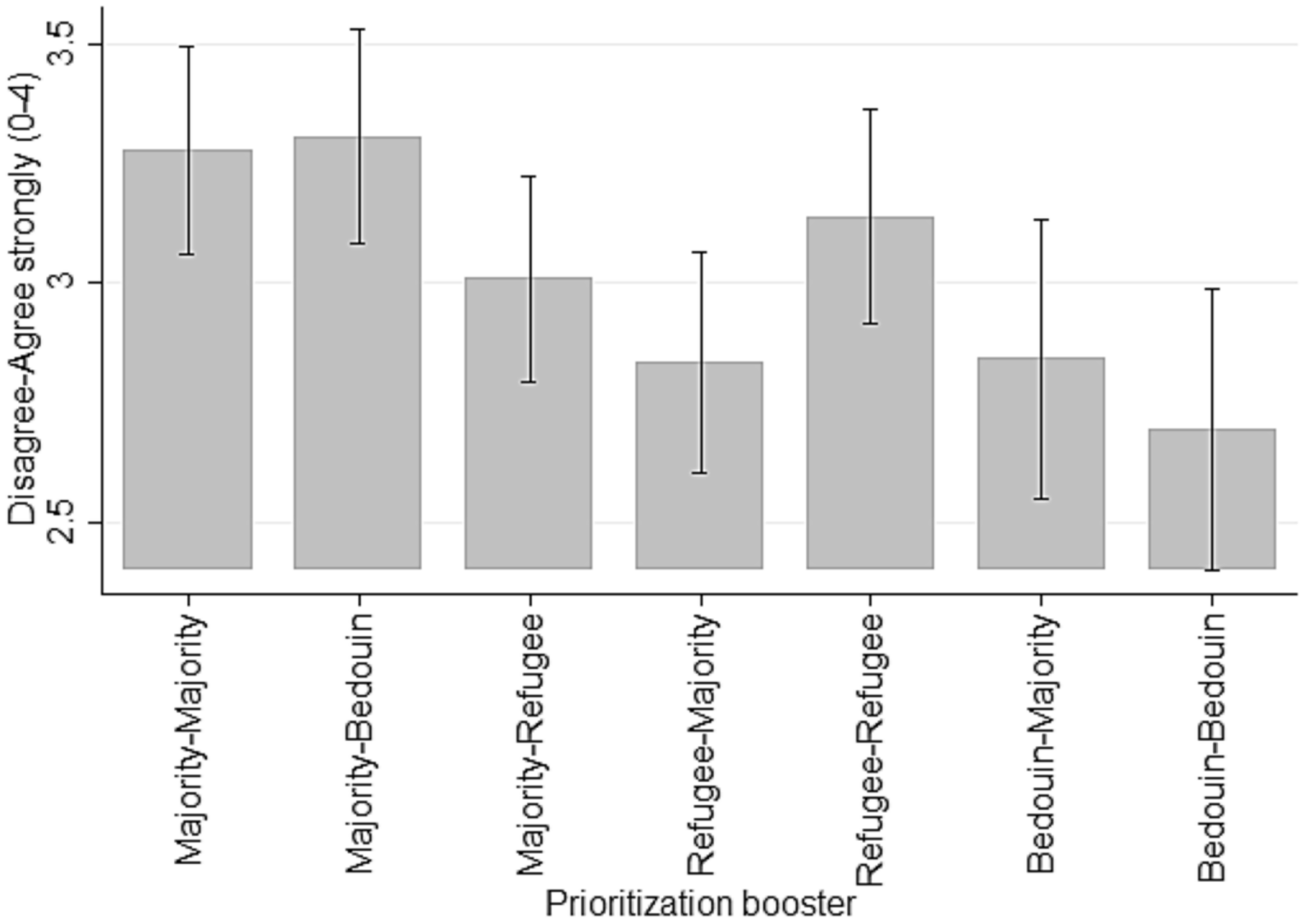
Deservingness of booster shot (marginal effects).

The survey experiment reveals three key results. First, Bedouins show significantly less in-group favoritism than refugees and majority, they make no significant difference between them and the majority. We observe the same result for majority-group members. They prioritize the booster for an older man belonging to their group as much as they support the booster for a Bedouin older man (Figure 1). However, Bedouins’ overall level of support for a prioritization of a majority-group member is significantly lower than the majority’s level of support for a prioritization of another majority-group member (*b* = −0.435; 95% CI: −0.799, −0.0705). Second, majority-group members support the prioritization of a booster for a refugee marginally less than for non-refugees (*b* = −0.267; 95% CI: −0.571, 0.0361). Third, refugees in turn prioritize majority-group members significantly less than the majority themselves (*b* = −0.443; 95% CI: −0.758, −0.127) and prioritize their own group more (*b* = 0.573; 95% CI: 0.131, 1.015).

Visible in a drop of significance levels, these differences are largely explained by socio-demographic variables such as sex and education, COVID-related variables, perceived discrimination, and religiosity (Table 2). These variables can explain how the majority thinks about the prioritization of an older refugee man, and how refugees evaluate the prioritization of another refugee. Moreover, Bedouin’s stance toward prioritization of a majority-group member can partly be explained by these variables.

While education makes surprisingly little difference, age goes along with more empathy toward an older man to be prioritized for the booster shot. This is visible in an increasing support of prioritization with age (*b* = 0.0115, *p* < 0.05). Moreover, we observe that inequality measured through perceived discrimination is significantly linked to the extent to which citizens support prioritization (*b* = −0.0417, *p* < 0.05). The more perceived discrimination, the lower the likelihood to support the prioritization of other groups. From the descriptive statistics (Table 1), we see that perceived discrimination is higher among minorities, particularly among Bedouins compared to majority-group members. This finding is significant (Bedouins *p* < 0.001, Refugees *p* < 0.05).

COVID-19 related factors matter only little if other factors are controlled for. Persons categorizing oneself to be at risk, are marginally more likely to prioritize an older man, thus showing more empathy. However, being fully vaccinated, or having experienced COVID-related deaths in the personal network was not significantly associated with the prioritization of an older man. Lastly, in line with theories suggesting higher levels of pro-sociality among religious individuals, we see that more religious individuals are marginally more in favor of prioritizing an older man than less religious individuals (*b* = 0.0474, *p* < 0.10). In addition, we see that religious minorities in Palestine (Christians/others) are significantly less likely to prioritize.

## Discussion

What have we learned from this study that we did not learn from other studies? As outlined at the beginning of this paper, the few studies that dedicated attention to citizenship found that immigrants were rated lower in the vaccine preference queue [e.g., (6, 9)]. However, we wanted to see if this pattern also extends to native minorities, as they might be perceived as an out-group as well. However, we find that Bedouins who constitute a salient minority within Palestine are not placed lower in the vaccine preference queue. Geographically and medically, many Bedouins live rather secluded (31), which might explain the lower level of prioritization of any group in the survey experiment.

However, Palestinian refugees tend to be placed a bit lower in the vaccine preference queue. This is a striking finding, as it underlines that the boundary runs along migration rather than citizenship or minority status. Even more striking is that the type of migration we see in Palestine arises primarily from internal displacement due to occupation and does not entail a different cultural background as it is the case for migration in the Western hemisphere. Hence, migration does matter, even on a regional level, and might further contribute to exclusion. Previous studies have shown that even internal migration can have consequences for socio-economic integration [e.g., (32)]. In a case study on the Balata camp, interviewees reported tensions with residents from surrounding areas, experiences of discrimination, isolation, and socio-economic cleavages (33, 34). On top of that, political tensions with the Israeli army frequently center on refugee camps with the situation escalating again shortly after we finished the field work [e.g., (35)].

While the role of religiosity in solidarity has been controversially discussed in prior research, we find that religious individuals tend to be more supportive of prioritization for booster shots overall. This is line with the higher benevolence found in previous studies [e.g., (14)] and it does not result from scepticism toward vaccination for their own group, as found, for instance, in the United States [e.g., (36)]. The question of why religiosity and spirituality have such fundamentally different cross-national effects on vaccination is an interesting endeavor for future research. A previous study using data from the United Kingdom argued that the relationship between spirituality and vaccination preferences was explained by a low trust into science (37). It is possible that higher levels of trust into Palestinian public institutions prevented the rise of skepticism toward vaccination among more religious individuals. However, we see denominational differences. Those not identifying as Sunni Muslims were significantly less likely to approve of the prioritization of an older man. It is possible that their status hampers their level of solidarity. Unfortunately, we were not able to estimate any interactions given their small sample size. Future research may use more scenarios and draw a larger sample in a representative fashion. Given the pandemic restrictions, the current study drew on a community-based sample and descriptive statistics should therefore be interpreted with caution given selection biases regarding an underrepresentation of female and illiterate Bedouins. Moreover, we have to keep in mind that sensitive questions (e.g., out-group attitudes, religion) are more prone to social desirability in face-to-face interviews. Atheists, for instance, might be less willing to identify themselves and express their views openly (38).

As this might have not been the last pandemic, and access to vaccination against COVID-19 will most likely remain a salient issue for the next years, this study has important societal implications. To change individual’s perceptions of deservingness, it would be first of all important to emphasize in the public debate that all residents need equal access to vaccination irrespective of their ethnic and social origin. Given the vulnerability of refugees and living circumstances making them more prone to contract the virus, we strongly recommend a prioritization of refugees from a humanitarian and empirical perspective. Palestinian refugees reported more deaths within the personal network (24). A prioritization for future vaccinations can help to protect refugees from more severe consequences.

In addition, perceived discrimination experiences among minorities, particularly among Bedouins are salient and can explain some of the majority-minority differences. Astonishingly, there was no in-group favoritism among Bedouins despite reporting higher levels of discrimination experiences. Nevertheless, on average, reduced discrimination in the public sphere but particularly the health sector will also likely affect the solidarity between citizens within and between groups. This deems the eradication of discrimination on grounds of origin to be relevant for future research and political implementation.

## Data availability statement

The raw data supporting the conclusions of this article will be made available by the authors, without undue reservation on request.

## Ethics statement

The studies involving humans were approved by the Research Ethics Committee of Al-Quds University (Ref No: 200/REC/2021). The studies were conducted in accordance with the local legislation and institutional requirements. The ethics committee/institutional review board waived the requirement of written informed consent for participation from the participants or the participants’ legal guardians/next of kin because interviews were conducted in the presence of guardians and verbal consent was seeked. Participants provided written consent.

## Author contributions

SC and AA designed the study and the questionnaire. SC drafted the manuscript and conducted the analyses. AA coordinated the data collection, wrote and commented on the draft. All authors contributed to the article and approved the submitted version.
