# Supplementary material for: COVID-19 booster prioritization in the West Bank: a survey experiment among Bedouins, refugees, and the majority group
Source: Front Public Health. 2023 Oct 4;11:1227559. doi: 10.3389/fpubh.2023.1227559 (PMC10582936; doi:10.3389/fpubh.2023.1227559)
Supplement: Supplementary file 1 [file Data_Sheet_1.PDF]

Questionnaire

**“Solidarity in Times of Uncertainty:
The Impact of COVID-19 on Minority- and
Majority-Group Members in Palestine”**

ARAB-GERMAN
YOUNG ACADEMY
OF SCIENCES AND
HUMANITIES

1. Have you heard about the "coronavirus" or COVID-19, an illness that causes fever, cough, and difficulty breathing?

- o No
- o Yes
- o Refuse
- o Don’t know

2. What was your main source of information during the pandemic?

- ❑ Government press briefings
- ❑ Government website on corona
- ❑ International organizations (WHO, UNICEF, etc.)
- ❑ Local TV stations
- ❑ International TV stations
- ❑ Internet
- ❑ Social media (e.g., facebook, Twitter)
- ❑ Health unit / health care worker
- ❑ Family members
- ❑ Neighbors or friends
- ❑ Employer, colleagues
- ❑ Religious leaders
- ❑ Campaigns on the street / door-to-door
- ❑ Other_______
- ❑ None
- ❑ Don’t know
- ❑ Refuse

3. How much do you trust the following persons and institutions in dealing with COVID-19?

	Completely distrust	Distrust	Neither distrust nor trust	Trust	Completely trust	Don't know	Refuse
Scientists	o	o	o	o	o	o	o
Local administration	o	o	o	o	o	o	o
Government	o	o	o	o	o	o	o
Health ministry	o	o	o	o	o	o	o
World Health Organization (WHO)	o	o	o	o	o	o	o
UN Health care	o	o	o	o	o	o	o
Governmental health care	o	o	o	o	o	o	o
Private health care	o	o	o	o	o	o	o
Religious leaders	o	o	o	o	o	o	o
Media	o	o	o	o	o	o	o
Police	o	o	o	o	o	o	o
Neighbors	o	o	o	o	o	o	o
Family members	o	o	o	o	o	o	o

4. Would you say that each of the following has done a very good, somewhat good, somewhat bad, or very bad job dealing with the COVID-19 outbreak?

	Very bad	Somewhat bad	Neither good nor bad	Somewhat good	Very good	Don't know	Refuse
Scientists	o	o	o	o	o	o	o
Local administration	o	o	o	o	o	o	o
Government	o	o	o	o	o	o	o

Health ministry	o	o	o	o	o	o	o
World Health Organization (WHO)	o	o	o	o	o	o	o
UN Health care	o	o	o	o	o	o	o
Governmental health care	o	o	o	o	o	o	o
Private health care	o	o	o	o	o	o	o
Religious leaders	o	o	o	o	o	o	o
Media	o	o	o	o	o	o	o
Police	o	o	o	o	o	o	o
Neighbors	o	o	o	o	o	o	o
Family members	o	o	o	o	o	o	o

5. To what extent do you see yourself as belonging to the Palestinian society?

- o Not at all
- o Hardly
- o Somewhat
- o Strongly
- o Very strongly
- o Don't know
- o Refuse

6. To what extent are you proud of being a member of the Palestinian society?

- o Not at all
- o Hardly
- o Somewhat
- o Strongly
- o Very strongly
- o Don't know
- o Refuse

7. What was your biggest worry during the pandemic?

____

____

8. *Interviewer instruction: The following question has different versions. Every respondent receives only one version randomly.*

For Bedouins: Please print half of the questionnaires using version A, and half of the questionnaires using version B.

Bedouins Group A: The outbreak of COVID-19 has placed an immense burden on societies and individuals who have become more isolated. Over the past months, countries have started to vaccinate its population against COVID-19. However, vaccines against COVID-19 are more effective with a booster shot. Imagine the case of a 70-year old Bedouin man. Do you think that he should be prioritized when the vaccination booster shots are given? Please answer on a scale from 1 "Disagree strongly" to 5 "Agree strongly".

o	o	o	o	o	o	o
Disagree strongly	2	3	4	Agree strongly	Don't know	Refuse

Bedouins Group B: The outbreak of COVID-19 has placed an immense burden on societies and individuals who have become more isolated. Over the past months, countries have started to vaccinate its population against COVID-19. However, vaccines against COVID-19 are more effective with a booster shot. Imagine the case of a 70-year old Palestinian man who has lived in his house his entire life. Do you think that he should be prioritized when the vaccination booster shots are given? Please answer on a scale from 1 "Disagree strongly" to 5 "Agree strongly".

o	o	o	o	o	o	o
Disagree strongly	2	3	4	Agree strongly	Don't know	Refuse

For Palestinian refugees: Please print half of the questionnaires using version A, and half of the questionnaires using version B.

Palestinian refugees Group A: The outbreak of COVID-19 has placed an immense burden on societies and individuals who have become more isolated. Over the past months, countries have started to vaccinate its population against COVID-19. However, vaccines against COVID-19 are more effective with a booster shot. Imagine the case of a 70-year old man who is a Palestinian refugee. Do you think that he should be prioritized when the vaccination booster shots are given? Please answer on a scale from 1 "Disagree strongly" to 5 "Agree strongly".

o	o	o	o	o	o	o
Disagree strongly	2	3	4	Agree strongly	Don't know	Refuse

Palestinian refugees Group B: The outbreak of COVID-19 has placed an immense burden on societies and individuals who have become more isolated. Over the past months, countries have started to vaccinate its population against COVID-19. However, vaccines against COVID-19 are more effective with a booster shot. Imagine the case of a 70-year old

Palestinian man who has lived in his house his entire life. Do you think that he should be prioritized when the vaccination booster shots are given? Please answer on a scale from 1 "Disagree strongly" to 5 "Agree strongly".

o	o	o	o	o	o	o
Disagree strongly	2	3	4	Agree strongly	Don't know	Refuse

For Palestinian majority-group: Please print 1/3 of the questionnaires using version A, 1/3 of the questionnaires using version B and 1/3 of the questionnaires using version C.

Palestinian majority group: Group A: The outbreak of COVID-19 has placed an immense burden on societies and individuals who have become more isolated. Over the past months, countries have started to vaccinate its population against COVID-19. However, vaccines against COVID-19 are more effective with a booster shot. Imagine the case of a 70-year old man who is a Palestinian refugee. Do you think that he should be prioritized when the vaccination booster shots are given? Please answer on a scale from 1 "Disagree strongly" to 5 "Agree strongly".

o	o	o	o	o	o	o
Disagree strongly	2	3	4	Agree strongly	Don't know	Refuse

Palestinian majority group: Group B: The outbreak of COVID-19 has placed an immense burden on societies and individuals who have become more isolated. Over the past months, countries have started to vaccinate its population against COVID-19. However, vaccines against COVID-19 are more effective with a booster shot. Imagine the case of a 70-year old Bedouin man. Do you think that he should be prioritized when the vaccination booster shots are given? Please answer on a scale from 1 "Disagree strongly" to 5 "Agree strongly".

o	o	o	o	o	o	o
Disagree strongly	2	3	4	Agree strongly	Don't know	Refuse

Palestinian majority group: Group C: The outbreak of COVID-19 has placed an immense burden on societies and individuals who have become more isolated. Over the past months, countries have started to vaccinate its population against COVID-19. However, vaccines against COVID-19 are more effective with a booster shot. Imagine the case of a 70-year old Palestinian man who has lived in his house his entire life. Do you think that he should be prioritized when the vaccination booster shots are given? Please answer on a scale from 1 "Disagree strongly" to 5 "Agree strongly".

o	o	o	o	o	o	o
Disagree strongly	2	3	4	Agree strongly	Don't know	Refuse

9. To what extent do you agree with the following statement on a scale from 1 "disagree strongly" to 5 "agree strongly": 'People in my community, regardless of nationality, ethnicity, and religion, are coming together to help each other during the current health crisis.'

o	o	o	o	o	o	o
Disagree strongly	2	3	4	Agree strongly	Don't know	Refuse

10. Have you experienced discrimination or been treated unfairly as a *Bedouin/Palestinian refugee/Palestinian [Interviewer adapt to group]* during the COVID-19 pandemic in the following places:

	No	Yes	Don't know	Refuse
Shops, bank or restaurant	o	o	o	o
Public areas such as parks and streets	o	o	o	o
Internet, social media	o	o	o	o
Work, job market	o	o	o	o
Public transport or taxis	o	o	o	o
School	o	o	o	o
Police	o	o	o	o
Housing	o	o	o	o
Courts	o	o	o	o
Border	o	o	o	o
Health care	o	o	o	o

11. Did you suffer from a lack of food during the pandemic?

o No
o Yes
o Don't know
o Refuse

12. If you suffered from a lack of food, how do you compare it to the time right before the COVID-19 situation started?
Please only ask if Q11=Yes

- o More
- o The same
- o Less
- o Don't know
- o Refuse

13. Which of the following measures have you used during the peaks of the COVID-19 pandemic? Please choose all answers that apply.

- ❑ I avoided crowded places
- ❑ I kept physical distance to other persons (at least 2 meters).
- ❑ I adjusted my schooling or work situation.
- ❑ I quarantined because of symptoms.
- ❑ I quarantined despite an absence of symptoms.
- ❑ I washed my hands more frequently and longer.
- ❑ I used disinfectants.
- ❑ I stocked up on water and/or food.
- ❑ I reduced face-to-face meetings and contact.
- ❑ I wore a face covering.
- ❑ Don't know
- ❑ Refuse

14. Do you have access to the following when you need it:

		Yes, always	Mostly	About half the time	Rarely	Never	Don't know	Refuse
1	Water	o	o	o	o	o	o	o
2	Soap	o	o	o	o	o	o	o

3	Sanitizer	o	o	o	o	o	o	o

15. In general, would you say your health is…

- o Very good
- o Good
- o Fair
- o Poor
- o Very poor
- o Don’t know
- o Refuse

16. Is your health in general now better, worse, or about the same as it was before the COVID-19 pandemic?

- o Better
- o Worse
- o The same
- o Don’t know
- o Refuse

17. During the COVID-19 pandemic, did you need to access the following health services? Please choose all answers that apply.

- ❑ Treatment \ care of chronic diseases
- ❑ Treatment \ care of non-chronic health conditions
- ❑ Buy Medicine \ Antibiotics
- ❑ Treatment or care of the disabled
- ❑ Post-natal or antenatal care
- ❑ Cancer treatment
- ❑ None of these
- ❑ Don’t know
- ❑ Refuse

18. If you needed to access health services, have those improved, worsened or stayed about the same during the COVID-19 pandemic?

Ask only if respondent chose at least one answer in Q17

- o Improved
- o The same
- o Worsened
- o Don’t know
- o Refuse

19. Did the situation with the COVID-19 pandemic ever result in you not getting health care when you needed it? Please select the answer that describes your situation best.

- Yes, because there was no available doctor or other good health provider
- Yes, because the wait times for the available doctor/health provider have become quite long
- Yes, because I was afraid to go out and get care due to COVID-19
- Yes, because I was not permitted to go out and get care due to the COVID-19 government restriction
- Yes, because my family prevented me from getting care due to worries about COVID-19
- The services or specialty I needed are not available where I live
- No, COVID-19 was not the issue
- No, I was able to get health care when needed
- Don’t know
- Refuse

20. Did the situation with the COVID-19 pandemic ever result in you not getting medication when you needed it?

- Yes, because the pharmacies and clinics were closed
- Yes, because even though the pharmacies and clinics are open, I was not able to get to them due to the social restrictions in place (e.g., curfew, lockdown, no buses)
- Yes, because I was afraid to go out and get medications due to COVID-19
- Yes, because my family did not allow me to go out because of the COVID-19 pandemic
- Yes, because even though the pharmacies and clinics are open, they do not have medications in supply
- No, COVID-19 was not the issue
- No, I was able to get medication when needed
- Don’t know
- Refuse

21. Over the last two weeks, how often have you been bothered feeling sad, down, depressed, or hopeless?

- Not at all (0 days out of the last 2 weeks)
- Several Days (1-6 days out of the last 2 weeks)
- More than half of the days (7-11 days out of the last 2 weeks)
- Nearly every day (12-14 days out of the last 2 weeks)
- Don’t know
- Refuse

22. Comparing the time right before the COVID-19 situation to now, have you felt sadder, less sad, or about the same?

- More
- The same
- Less
- Don't know
- Refuse

23. Which of the following describes how you are coping and responding to COVID-19? Are you… (Please choose all answers that apply)

- ❑ Getting angry more quickly
- ❑ Arguing more often
- ❑ Praying more often
- ❑ Helping household members more with chores or other tasks
- ❑ Fearing and worrying about your own health and the health of your loved ones
- ❑ None of these
- ❑ Don't know
- ❑ Refuse

24. Compared to before the Covid-19 situation, has physical violence experienced in your community increased, decreased, or stayed the same?

- Increased
- The same
- Decreased
- Don't know
- Refuse

25. Have you smoked during the COVID-19 pandemic?

- No
- Yes
- Don't know
- Refuse

26. Comparing the time right before the COVID-19 situation to now, are you smoking more, less, or about the same number of cigarettes as usual?

If Q25 = No → Q26 = "The same"

- More

- The same
- Less
- Don’t know
- Refuse

27. Have you experienced any of the following symptoms during the COVID-19 pandemic? Please choose all answers that apply.

- ❑ Fever
- ❑ Dry cough
- ❑ Shortness of breath or difficulty breathing
- ❑ Loss of taste or smell
- ❑ Chills or repeated shaking with chills
- ❑ Muscle pain, headache, or sore throat
- ❑ Diarrhea
- ❑ None of these
- ❑ Don’t know
- ❑ Refuse

28. Have you been tested positive for COVID-19?

- No
- Yes
- Don’t know
- Refuse

29. Which kind of vaccine have you taken?

- Moderna
- Pfizer/ Biontech
- Sputnik
- Sinovac
- Other______

30. Have you been fully vaccinated against COVID-19?

- No
- Yes
- Don’t know
- Refuse

31. If you have not been vaccinated yet, what was the reason? Please choose all answers that apply.

- ❑ The chances of me catching COVID-19 are low
- ❑ The chances of me becoming seriously unwell from COVID-19 are low
- ❑ The impact of COVID-19 is being greatly exaggerated
- ❑ Vaccines are limited and other people need it more than me
- ❑ Herd immunity will protect me even if I don't have the vaccine
- ❑ I don't think I would be offered the vaccine for free and I wouldn't pay for it
- ❑ I don't think it would be effective at stopping me catching COVID-19
- ❑ I am worried about side effects
- ❑ I am worried about unknown future effects of the vaccine
- ❑ I am pregnant
- ❑ I don't trust vaccines
- ❑ I have a condition which would make it unsafe for me
- ❑ I cannot get to the vaccination centre (safely)
- ❑ Because of my religion
- ❑ None of these
- ❑ Don't know
- ❑ Refuse

32. Do you count as an at-risk group for COVID-19-complications (This means you may be older and/or suffer from an illness that makes you more vulnerable)?

- o No
- o Yes
- o Don't know
- o Refuse

33. Did you know anyone who died of COVID–19 in person?

- o No
- o Yes
- o Don't know
- o Refuse

34. What is your gender?

- o Male
- o Female
- o Don't know
- o Refuse

35. What is your year of birth? Please indicate 4 digits.

____

36. To which of the following devices do you have access to?

❑ TV
❑ Computer
❑ Tablet
❑ Mobile phone
❑ Radio
❑ Other____________
❑ None
❑ Don’t know
❑ Refuse

37. Do you have internet access at the place you are residing?

- No
- Yes
- Don’t know
- Refuse

38. What is your highest level of education?

- Illiterate, no formal education
- Elementary School
- Secondary school
- Higher non university education / vocational training
- Bachelor
- Master
- PhD
- Don’t know
- Refuse

39. What is your current employment status?

- Full-time employment
- Part-time employment
- Housewife/ househusband
- Retired/ invalidity pensioner
- Ill health/disability
- Unemployed/ seeking work
- Student (full-time)
- Parental leave

- Caring for family member
- Don’t know
- Refuse

40. Has your employment situation improved, worsened or stayed about the same during the COVID-19 pandemic?

- Improved
- The same
- Worsened
- Don’t know
- Refuse

41. Are you a beneficiary of any of the following social assistance programs? Please choose all answers that apply.

- ❑ Food \ food vouchers
- ❑ Free medicine \ health treatment
- ❑ Government cash transfer program
- ❑ Job opportunities
- ❑ Martyrs/injuries compensation
- ❑ Assistance from private non-profit organizations
- ❑ Assistance from UNRWA
- ❑ Family / friends transfer
- ❑ Other________
- ❑ Don’t know
- ❑ Refuse

42. What is your relationship status?

- Married
- Single
- Engaged
- Divorced
- Widowed
- Don’t know
- Refuse

43. Do you live with any of the following persons?

- ❑ Partner
- ❑ Parents
- ❑ Children
- ❑ Siblings

- ❑ Grandchildren
- ❑ Other relatives
- ❑ Other persons___________
- ❑ Don’t know
- ❑ Refuse

44. How many children do you have?

____

- o Don’t know
- o Refuse

45. Do you think about emigrating from your country?

- o No
- o Yes
- o Don’t know
- o Refuse

46. If you consider emigrating, where would you most likely emigrate to?

Ask only if Q45=Yes

- o ___________________
- o Don’t know
- o Refuse

47. What is your religious denomination?

- o Sunni
- o Catholic
- o Protestant
- o Christian Orthodox
- o None
- o Other_______
- o Don’t know
- o Refuse

48. Regardless of whether you belong to a particular religion, how religious would you say you are on a scale from 0 “not religious at all” to 10 “very religious”?

0 Not religious at all	1	2	3	4	5	6	7	8	9	10 very religious	Don't know	Refuse

We have come to the end of our interview. Thank you very much for participating in this study. We would like to keep you informed about the outcome of this study, if you agree. Please share your email address or telephone number with the interviewer to stay informed. Your personal data will be kept strictly confidential and stored separate from your answers in this interview. You can withdraw your contact details at any time.

Phone number:

E-Mail address:

*[Interviewer note governorate and camp if applicable]*____________________
